# Subvalvular Aortic Stenosis: Current Insights into an Often Overlooked Cause of Left Ventricular Outflow Tract (LVOT) Obstruction

**DOI:** 10.3390/medsci14050515

**Published:** 2026-08-25

**Authors:** Vasileios Leivaditis, Athanasios Papatriantafyllou, Francesk Mulita, Vasiliki Androutsopoulou, Elias Liolis, Konstantinos Tasios, Andreas Antzoulas, Dimitrios Litsas, Theodora Skoura, Konstantinos Nikolakopoulos, Spyros Papadoulas, Nikolaos G. Baikoussis

**Affiliations:** 1 Department of Cardiothoracic and Vascular Surgery, Westpfalz Klinikum, 67655 Kaiserslautern, Germany; 2 Second Department of Surgery, Medical School, Democritus University of Thrace, 68100 Alexandroupolis, Greece; 3 Department of Cardiothoracic Surgery, Faculty of Medicine, University of Thessaly, Biopolis, 41110 Larissa, Greece; androutsopoulouvasiliki@uth.gr; 4 Department of Oncology, General University Hospital of Patras, 26504 Patras, Greece; 5 John Radcliffe Hospital Emergency Department, University Hospitals NHS Foundation Trust, Headley Way, Headington, Oxford OX3 9DU, UK; 6 Department of Surgery, General University Hospital of Patras, 26504 Patras, Greece; 7 Department of Surgery, General Hospital of Lamia, 35100 Lamia, Greece; 8 Medical School, National and Kapodistrian University of Athens (NKUA), Aretaeion Hospital, 11528 Athens, Greece; tskoura@yahoo.com; 9 Department of Vascular Surgery, University of Patras, 26504 Patras, Greece; 10 Department of Cardiac Surgery, Ippokrateio General Hospital of Athens, 11527 Athens, Greece

**Keywords:** subaortic stenosis, aortic valve stenosis, subvalvular aortic stenosis, Shone syndrome, subaortic membrane, aortic valve replacement, LVOT, interventricular septum hypertrophy, heart valve surgery

## Abstract

Background: Subvalvular aortic stenosis (SAS) is an important cause of fixed left ventricular outflow tract obstruction and the second most common form of aortic stenosis. Although traditionally considered a congenital lesion, increasing evidence suggests that SAS often behaves as a progressive condition influenced by anatomical and hemodynamic factors. The disease may lead to significant complications, including left ventricular hypertrophy and progressive aortic regurgitation. Materials and Methods: A narrative review of the current literature was performed to summarize contemporary knowledge regarding the epidemiology, pathophysiology, clinical presentation, diagnostic evaluation, and management of subvalvular aortic stenosis. Relevant studies were identified through searches of major medical databases and were critically analyzed to provide an overview of current concepts and clinical practice. Results: Subvalvular aortic stenosis demonstrates considerable anatomical and clinical heterogeneity, ranging from discrete subaortic membranes to fibromuscular tunnel-type obstruction. The condition often progresses over time, with increasing LVOT gradients and a high incidence of associated aortic regurgitation. Echocardiography remains the primary diagnostic modality, while advanced imaging techniques provide additional anatomical detail and assist in surgical planning. Surgical resection remains the cornerstone of treatment when significant obstruction or related complications develop. Conclusions: Subvalvular aortic stenosis is a complex and progressive disease requiring careful imaging assessment and timely surgical management. Although surgical outcomes are generally excellent, recurrence and progressive aortic valve involvement remain important long-term considerations. Ongoing research aimed at improving risk stratification and optimizing surgical strategies may further enhance patient outcomes.

## 1. Introduction

Subvalvular aortic stenosis (SAS) is a distinctive form of fixed left ventricular outflow tract obstruction characterized by progressive narrowing below the aortic valve. Although less common than valvular aortic stenosis, it represents the second most frequent cause of aortic stenosis and accounts for a substantial proportion of LVOT obstructive lesions [1,2,3,4]. In most patients, the obstruction is caused by a discrete subaortic membrane; in roughly 70% of cases, an isolated membrane is identified, which may progress to a more complex, tunnel-like narrowing of the LVOT [5,6]. The anatomical spectrum of SAS is heterogeneous and includes a thin, crescent-shaped membrane immediately beneath the aortic valve, a thicker fibromuscular ridge, or a longer tubular (tunnel) fibromuscular channel extending along the LVOT, occurring either in isolation or in combination [7,8,9,10].

SAS is typically not diagnosed in infancy; rather, it commonly becomes clinically apparent during the first decade of life as a progressive fixed LVOT obstruction associated with left ventricular hypertrophy (LVH) and, frequently, aortic regurgitation (AR) [11,12]. Increasing evidence suggests that SAS should not be viewed as a purely congenital abnormality. The delayed onset of clinical manifestations, progressive increase in obstruction over time, and frequent recurrence after surgical resection support the concept of an evolving disease process driven by both anatomical predisposition and hemodynamic factors [13,14,15,16]. Recent contemporary evidence further supports this concept, highlighting that disease progression results from the interaction between patient-specific LVOT anatomy, abnormal flow dynamics, and progressive fibromuscular remodeling rather than from a static congenital lesion alone [17,18]. For this reason, awareness of its anatomical variants and mechanisms of progression is clinically important, as lesion morphology influences both surgical strategy and the risk of recurrence.

The lesion most often consists of a membrane attached to the ventricular septum, sometimes partially or circumferentially encircling the LVOT and producing a fixed obstruction [19,20,21,22,23,24,25,26]. The membrane may be located immediately beneath the aortic valve or further apically within the left ventricle, and it can extend toward the aortic annulus. In some patients, subaortic tissue involves the basal portions of the aortic valve cusps, restricting cusp mobility and promoting or worsening AR through chronic leaflet trauma and altered flow dynamics [13,14,15,16,17,18,19]. Overall, the clinical course is usually gradual and progressive rather than static, with deterioration driven by increasing obstruction and secondary valvular involvement [15,16,17,18,19,20].

Importantly, SAS is rarely an isolated finding. It is frequently associated with other congenital cardiac abnormalities, including ventricular septal defect, patent ductus arteriosus, coarctation of the aorta, bicuspid aortic valve, abnormal papillary muscle anatomy, and atrioventricular septal defect, among others [5,6,7,8,9,13]. The presence of associated congenital lesions may substantially influence disease progression, operative complexity, and long-term outcomes, emphasizing the importance of comprehensive anatomical assessment and individualized surgical planning [17,27,28]. Consequently, SAS is often detected incidentally during evaluation or follow-up of patients with concomitant congenital heart disease [13,14].

Despite the growing literature on SAS, important challenges remain in translating its evolving pathophysiological concepts and heterogeneous evidence into practical clinical decision-making. Previous reviews have provided valuable overviews of the disease and, more recently, its multimodality imaging and management [17,28]; however, uncertainties persist regarding the interpretation of disease progression, selection and integration of imaging modalities, thresholds for surgical intervention, choice of operative strategy according to anatomical subtype, and predictors of postoperative recurrence. The present narrative review therefore aims to provide an updated, clinically oriented synthesis that integrates these elements across the continuum of care, from diagnosis and risk stratification to surgical management and lifelong surveillance. Intended for cardiologists, cardiac surgeons, congenital heart disease specialists, and clinicians involved in longitudinal follow-up, the review places particular emphasis on translating the available evidence into practical considerations for individualized management while highlighting areas in which recommendations remain dependent on observational evidence and expert consensus.

**Figure 1:**
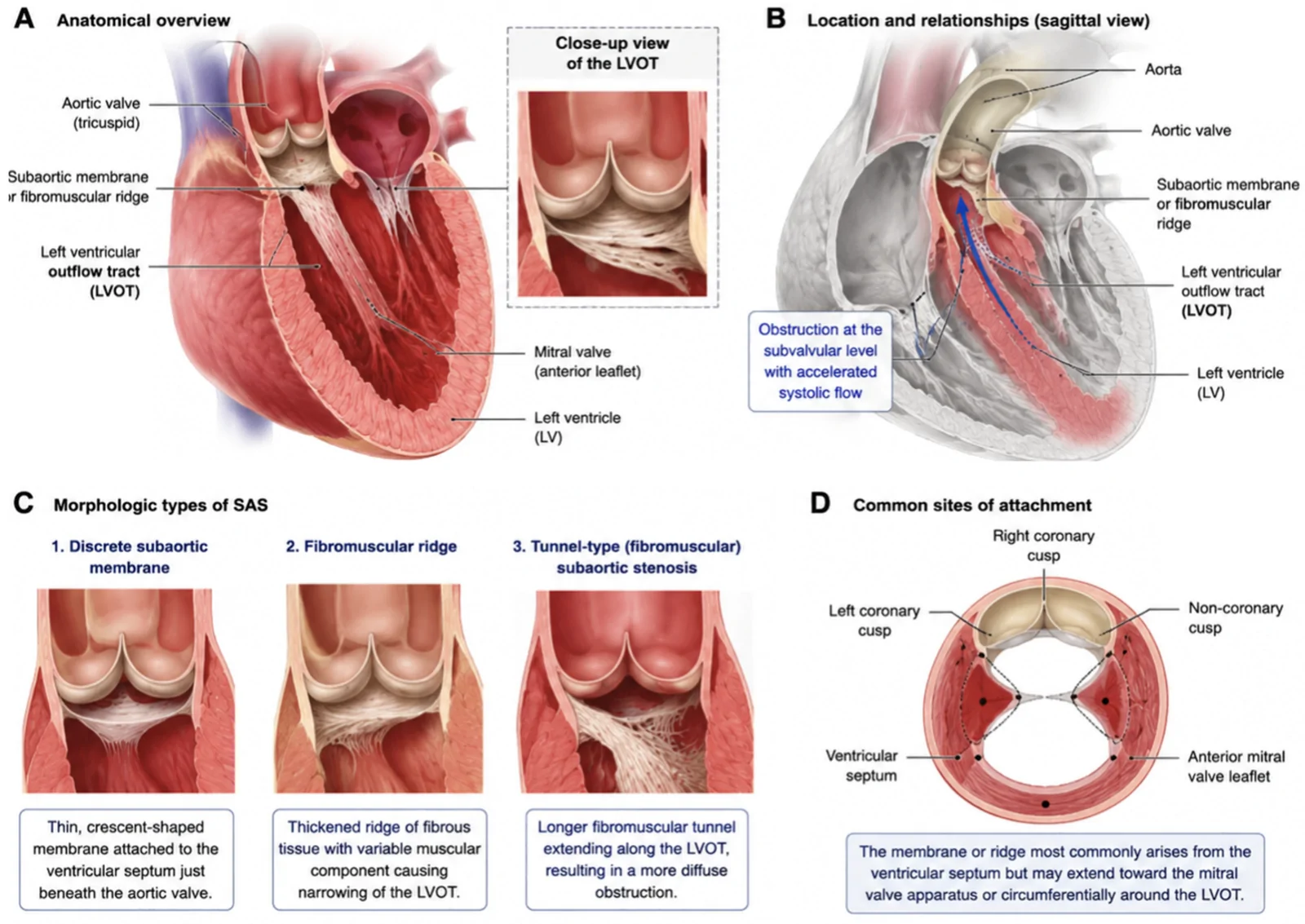
Anatomical spectrum of subvalvular aortic stenosis (SAS). (**A**) Schematic anatomical overview of the left ventricular outflow tract (LVOT) demonstrating the typical location of subvalvular obstruction immediately beneath the aortic valve. The illustration highlights the anatomical relationship between the subaortic lesion, aortic valve, interventricular septum, LVOT, and left ventricle. (**B**) Representative morphological variants of SAS. From left to right: discrete subaortic membrane, fibromuscular ridge, tunnel-type subaortic stenosis, and circumferential/complex subvalvular obstruction. These anatomical patterns differ in lesion extent, degree of LVOT narrowing, and surgical complexity. (**C**) Typical echocardiographic appearance of SAS on transthoracic echocardiography, demonstrating the visualization of the subaortic membrane and associated turbulent systolic flow across the LVOT. (**D**) Examples of multimodality imaging used for anatomical characterization and preoperative assessment, including transesophageal echocardiography, cardiac magnetic resonance imaging, and three-dimensional computed tomography, illustrating the complementary role of advanced imaging techniques in defining lesion morphology and surgical planning.

The principal anatomical characteristics and morphological spectrum of subvalvular aortic stenosis are summarized in Figure 1, illustrating the location of the obstruction within the left ventricular outflow tract and the most common anatomical variants encountered in clinical practice.

## 2. Materials and Methods

This narrative review was conducted to provide a clinically oriented synthesis of the available evidence regarding subvalvular aortic stenosis (SAS), with particular emphasis on epidemiology, pathophysiology, natural history, clinical presentation, multimodality imaging, therapeutic management, surgical outcomes, recurrence, and long-term surveillance. A structured literature search was performed in the PubMed/MEDLINE and Scopus databases for publications from January 2000 through May 2026, with the final search conducted on 6 June 2026.

The search strategy combined the terms “subaortic stenosis”, “subvalvular aortic stenosis”, “discrete subaortic stenosis”, and “subaortic membrane” with relevant terms including “epidemiology”, “pathophysiology”, “natural history”, “diagnosis”, “echocardiography”, “cardiac magnetic resonance”, “computed tomography”, “surgery”, “surgical treatment”, “outcomes”, “recurrence”, and “aortic regurgitation”. Boolean operators (AND/OR) were used as appropriate to combine search terms. Reference lists of relevant original studies and review articles were additionally screened to identify publications not retrieved through the electronic database search. Although the predefined search period began in January 2000, a limited number of landmark publications from the 1990s identified through reference screening were also included when they provided foundational evidence regarding the natural history, pathophysiology, or clinical management of SAS that remained relevant to the present review.

Eligible publications included original clinical studies, prospective and retrospective cohort studies, observational studies, imaging studies, surgical series, guideline and consensus documents, and relevant review articles addressing the epidemiology, natural history, pathophysiology, diagnosis, treatment, or long-term outcomes of SAS. Particular consideration was given to studies providing clinically relevant information regarding disease progression, multimodality imaging, indications for intervention, operative techniques, postoperative recurrence, aortic valve involvement, and long-term follow-up. Case reports, conference abstracts without full-text publication, non-English-language publications, and studies without specific relevance to SAS were excluded.

The structured database search identified 281 records, of which 166 remained after removal of duplicates. Following title and abstract screening, 94 publications underwent full-text assessment. After application of the eligibility criteria and supplementary reference screening, 67 publications were considered relevant and included in the final narrative synthesis, including the selected landmark publications predating 2000. Study selection was performed independently by two reviewers. Any differences regarding eligibility or relevance were resolved through discussion and consensus.

**Figure 2:**
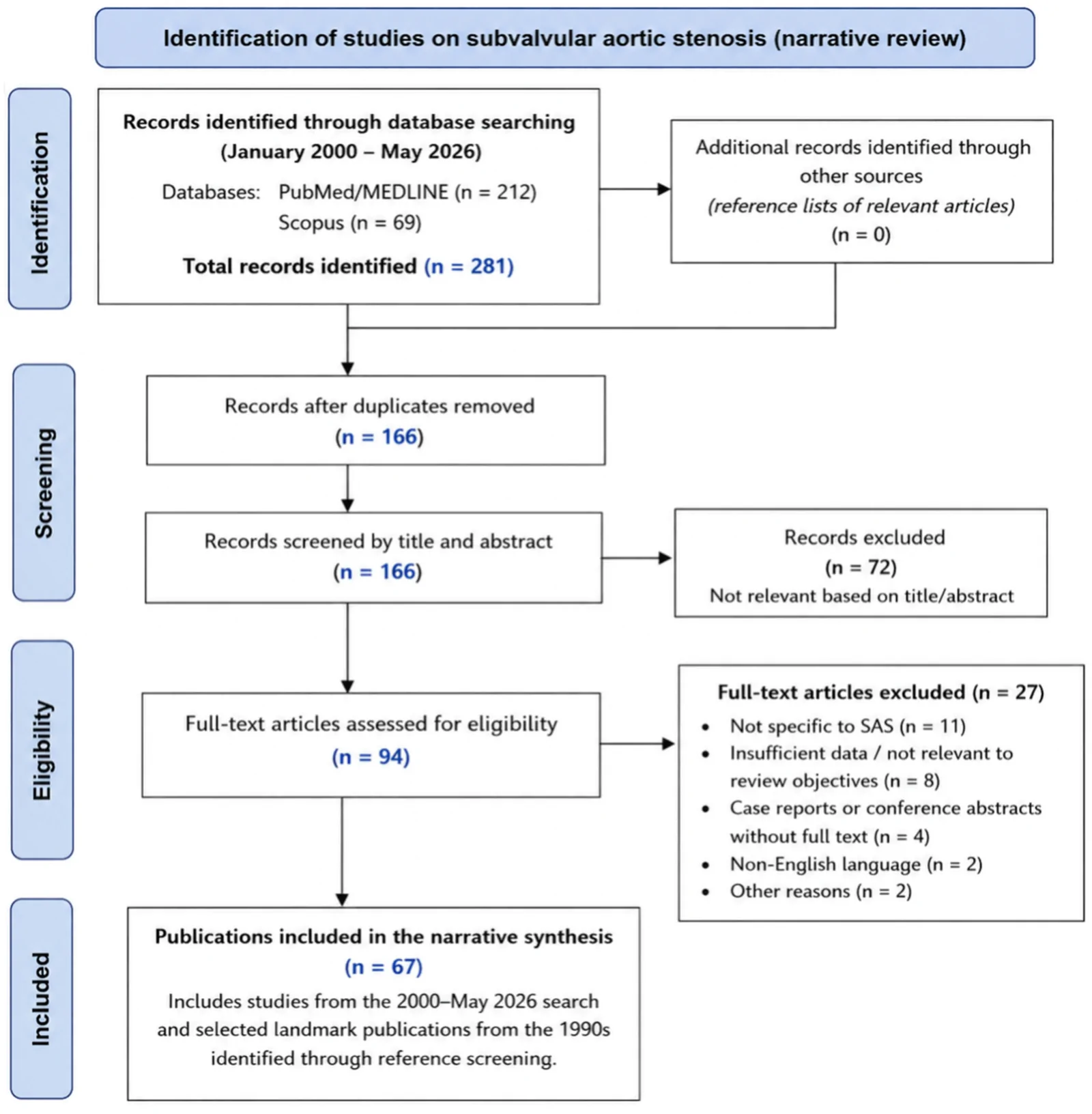
Flow diagram of the literature identification and selection process.

Given the heterogeneity of the available literature with respect to patient populations, anatomical phenotypes, imaging approaches, surgical strategies, outcome definitions, and duration of follow-up, the evidence was synthesized narratively rather than quantitatively. The review was not designed as a systematic review, scoping review, or meta-analysis, and formal quantitative pooling was therefore not performed. The preparation and reporting of the manuscript were guided by the principles of the Scale for the Assessment of Narrative Review Articles (SANRA), with particular attention to justification of the review, transparency of the literature search, appropriate referencing, scientific reasoning, and presentation of clinically relevant evidence [29]. Accordingly, PRISMA/PRISMA-ScR reporting was not applied. The literature identification and selection process, including database retrieval, duplicate removal, title and abstract screening, full-text assessment, and final inclusion in the narrative synthesis, is summarized in Figure 2.

## 3. Pathophysiology and Mechanisms of Disease Development

Subvalvular aortic stenosis (SAS) is a complex and multifactorial pathological entity characterized by the development of a fixed obstruction within the left ventricular outflow tract (LVOT). Unlike valvular aortic stenosis, SAS arises below the level of the aortic valve and involves abnormal fibromuscular tissue proliferation that progressively narrows the outflow tract. Multiple interacting mechanisms have been implicated in its pathogenesis, including genetic susceptibility, abnormal LVOT anatomy, and disturbed intracardiac flow dynamics [13,30,31,32]. Rather than representing a single congenital malformation, SAS is increasingly recognized as a dynamic disease process influenced by both structural predisposition and hemodynamic forces.

Several anatomical configurations of the LVOT appear to predispose patients to the development of SAS. These include a relatively narrow LVOT, exaggerated aortic override, increased mitral–aortic septation, and a steep atrioventricular septal angle, all of which create unfavorable flow patterns within the outflow tract [1,13,19]. Such configurations promote flow acceleration and turbulence during systole, particularly along the interventricular septum. Importantly, these anatomical features may be subtle and not necessarily obstructive at birth, which helps explain why SAS is often absent in neonates and early infancy despite being classified as a congenital heart disease. Recent multimodality imaging studies have further demonstrated that subtle geometric abnormalities of the LVOT may precede clinically significant obstruction, supporting the concept that anatomical configuration and abnormal flow interact throughout disease evolution rather than representing isolated phenomena [17].

Chronic exposure to turbulent blood flow leads to persistently increased shear stress on the endocardial surface of the interventricular septum. This mechanical stress induces endothelial dysfunction and triggers a cascade of cellular and molecular responses, including activation of fibroblasts and smooth muscle cells, increased expression of growth factors, and abnormal extracellular matrix remodeling. Over time, these processes result in progressive fibromuscular proliferation and collagen deposition, giving rise to a discrete subaortic membrane or, in more advanced cases, a fibromuscular ridge or tunnel-like narrowing of the LVOT [1,13,25]. Histopathological studies support this mechanism, demonstrating dense, irregular collagen fibers with sparse cellularity, consistent with a chronic proliferative response rather than an embryological remnant. Current evidence suggests that these pathological changes are maintained by persistent hemodynamic stimulation, explaining why disease progression may continue even after prolonged periods of clinical stability and why recurrence remains possible following apparently complete surgical resection [17].

These observations have led to the widely accepted hypothesis that SAS, although traditionally considered a congenital lesion, often behaves as a progressive or even acquired condition. The rarity of SAS at birth, its gradual progression during childhood or early adulthood, and its tendency to recur after surgical resection all support the notion that ongoing hemodynamic stimuli play a central role in disease evolution rather than a fixed developmental anomaly present from birth [13,14].

In addition, surgical correction of associated congenital heart defects may itself contribute to the later development or progression of SAS. Procedures that alter LVOT geometry or redirect intracardiac flow—such as ventricular septal defect closure or repair of other left-sided obstructive lesions—can increase flow velocity and turbulence within the LVOT, thereby enhancing shear stress on the interventricular septum and promoting fibromuscular proliferation [13]. This phenomenon underscores the importance of long-term surveillance in patients who have undergone repair of congenital heart disease, even when initial postoperative results are satisfactory. Secondary or recurrent LVOT obstruction may develop years after correction of associated congenital defects, emphasizing the importance of recognizing postoperative geometric changes as an additional mechanism contributing to disease progression [33,34].

Rarely, abnormalities of the papillary muscles or mitral valve apparatus have been described as an additional mechanism of LVOT obstruction. These anomalies may displace or tether the mitral valve, encroach upon the LVOT, or generate abnormal flow vectors that exacerbate turbulence. In some cases, this presentation may closely resemble hypertrophic cardiomyopathy, complicating both diagnostic evaluation and therapeutic decision-making [35,36,37,38]. Furthermore, isolated reports have highlighted that membranous SAS may coexist with hypertrophic cardiomyopathy, creating diagnostic uncertainty and reinforcing the importance of comprehensive multimodality imaging before surgical intervention [39,40]. Recognition of these less common mechanisms is clinically important, as they may influence surgical planning and explain suboptimal outcomes if not adequately addressed.

Overall, understanding the multifactorial pathophysiology of SAS is essential for appropriate clinical management. The interplay between anatomical predisposition, altered flow dynamics, and progressive fibromuscular response explains not only the variability in disease presentation and progression but also the significant risk of recurrence after surgical intervention. These mechanisms directly inform surgical strategy, timing of intervention, and the need for meticulous long-term follow-up.

**Figure 3:**
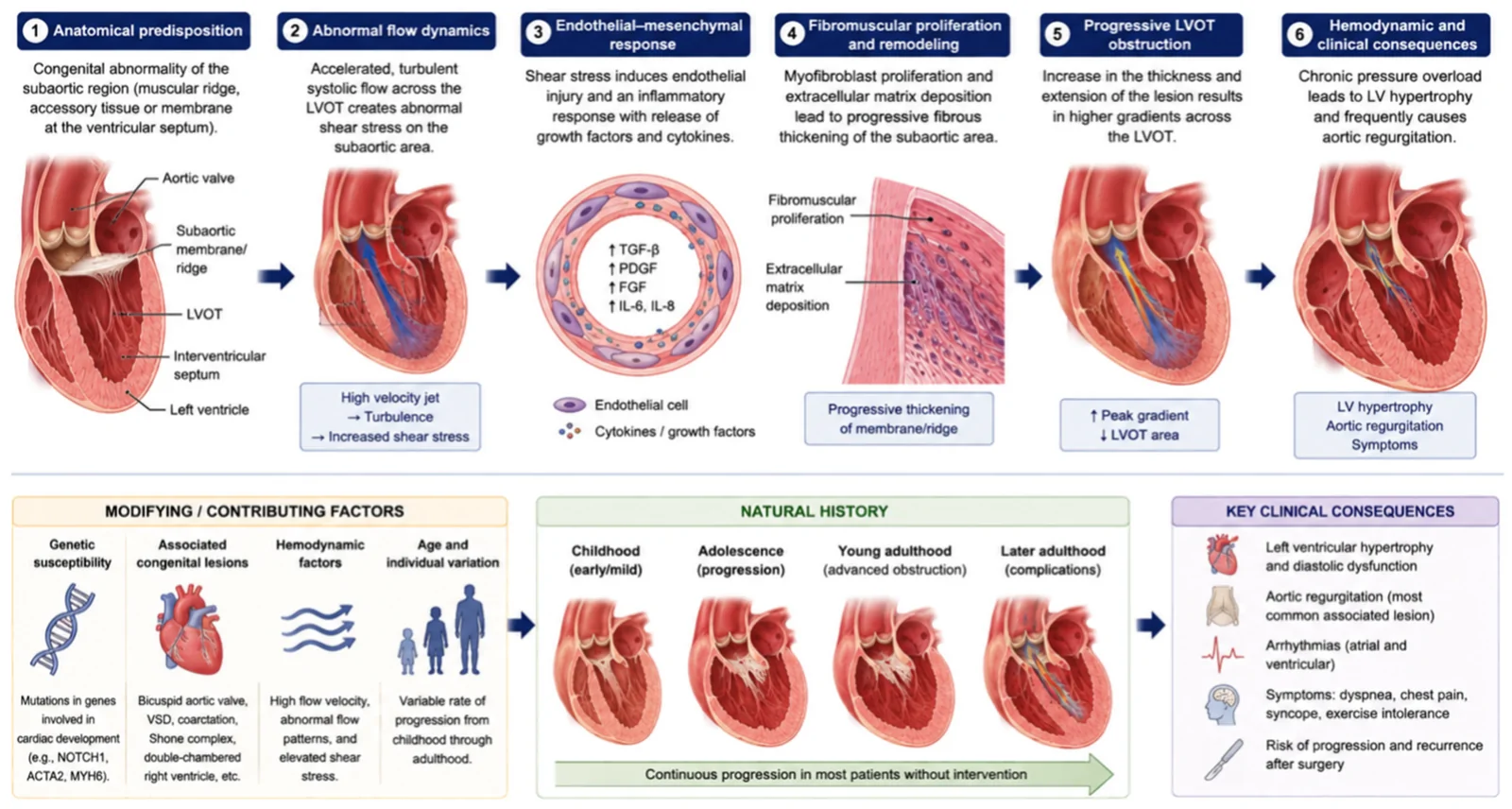
Proposed mechanisms underlying the development and progression of subvalvular aortic stenosis. Schematic overview illustrating the interplay between anatomical predisposition, abnormal left ventricular outflow tract flow dynamics, endothelial injury, progressive fibromuscular remodeling, and increasing left ventricular outflow tract obstruction. The figure also summarizes the major factors influencing disease progression, the natural history of subvalvular aortic stenosis, and its principal hemodynamic and clinical consequences, including left ventricular hypertrophy, aortic regurgitation, and the risk of postoperative recurrence.

The currently accepted pathophysiological mechanisms underlying the development and progression of subvalvular aortic stenosis are summarized in Figure 3, highlighting the interaction between anatomical predisposition, abnormal flow dynamics, progressive fibromuscular remodeling, and their subsequent hemodynamic and clinical consequences. The principal morphological variants of subvalvular aortic stenosis and their distinguishing anatomical, clinical, and surgical characteristics are summarized in Table 1.

## 4. Epidemiology and Associated Congenital Heart Disease

Although SAS is relatively uncommon in neonates and young infants, it represents the second most common form of aortic stenosis across all age groups. Overall, SAS accounts for approximately 1% of all congenital heart defects and for 15–20% of fixed LVOT obstructive lesions [13]. Although SAS is relatively uncommon in neonates and young infants, it represents an important cause of fixed LVOT obstruction in children and young adults and is generally regarded as the second most frequent form of aortic stenosis in this population. Its delayed clinical manifestation, often several years after birth, supports the concept of a progressive lesion rather than a purely congenital fixed obstruction present at birth [41,42,43,44].

A male predominance has been reported in pediatric SAS, with several series demonstrating a male-to-female ratio approaching 2:1 [44,45,46]. However, the magnitude of this sex difference varies among cohorts and appears less pronounced in adult populations; for example, Oliver et al. reported that 52% of 134 adults with discrete SAS were male [3]. The reasons for this sex disparity remain unclear but may reflect sex-related differences in cardiac anatomy, growth patterns, or susceptibility to flow-related endothelial injury. Regardless of the underlying mechanism, this epidemiological feature is important for risk stratification and clinical suspicion, particularly in pediatric and adolescent patients presenting with LVOT obstruction. Adult cohorts have similarly demonstrated a persistent male predominance, indicating that this sex distribution remains consistent across different age groups [17].

In the adult population, SAS accounts for approximately 6.5% of all congenital heart diseases, highlighting that it is not exclusively a pediatric condition and may remain undiagnosed until adulthood. Adult presentations are often discovered incidentally or during evaluation for symptoms related to progressive LVOT obstruction, aortic regurgitation, or associated aortic pathology [3]. Approximately one-quarter of surgical procedures for SAS are now performed in adults, reflecting improved recognition of late-presenting disease and the progressive nature of the condition [17,18].

Notably, SAS is rarely an isolated anomaly. It is associated with other congenital cardiac malformations in approximately 50–65% of patients [5,11,13]. The most frequently reported associated lesions include ventricular septal defect, patent ductus arteriosus, pulmonic stenosis, coarctation of the aorta, bicuspid aortic valve, and abnormalities of the mitral valve apparatus or papillary muscles [5,13]. Less frequently, SAS has also been reported in association with complex congenital syndromes, including Shone syndrome, Noonan syndrome, and congenital rubella syndrome [17]. The frequent coexistence of these anomalies suggests shared developmental or hemodynamic pathways and has important implications for both diagnosis and management. These associations reinforce the necessity of comprehensive anatomical evaluation at the time of diagnosis and justify long-term surveillance, as the presence of concomitant defects may influence disease progression, surgical complexity, and postoperative outcomes.

## 5. Clinical Presentation and Symptoms

The clinical presentation of SAS is variable and largely depends on the severity of LVOT obstruction, the rate of disease progression, and the presence of associated cardiac abnormalities. Exertional dyspnea is the most common presenting symptom, reported in approximately 40% of symptomatic patients. It primarily reflects elevated left ventricular filling pressures caused by impaired diastolic relaxation and reduced compliance of the hypertrophied left ventricle [13,36]. As diastolic dysfunction progresses, pulmonary venous congestion may develop, leading to exercise intolerance and, in advanced cases, symptoms of heart failure. The onset of symptoms is frequently delayed despite progressive anatomical obstruction, emphasizing the limited correlation between symptom burden and disease severity in many patients [17].

In the presence of severe LVOT obstruction, the fixed limitation to systolic ejection may result in an inadequate increase in cardiac output during physical activity. This can lead to systemic hypotension and reduced cerebral perfusion, manifesting clinically as exertional dizziness, presyncope, or syncope. In pediatric patients, true syncope is relatively uncommon and, when present, should prompt careful evaluation for associated arrhythmias or conduction disturbances rather than being attributed solely to mechanical obstruction [16,38,41].

Angina pectoris occurs in up to 25% of symptomatic patients and is the consequence of a mismatch between myocardial oxygen supply and demand [13,37]. The hypertrophied left ventricle has increased oxygen requirements, while coronary perfusion may be compromised by elevated intraventricular pressures and reduced aortic diastolic pressure, particularly in the presence of associated aortic regurgitation. Importantly, angina may occur even in the absence of epicardial coronary artery disease, especially in younger patients. In adults, symptoms may occasionally mimic hypertrophic obstructive cardiomyopathy or valvular aortic stenosis, making careful anatomical evaluation essential for establishing the correct diagnosis [39,47,48,49].

Despite these potential manifestations, a substantial proportion of patients with SAS remain asymptomatic for prolonged periods, even in the presence of significant LVOT gradients. This asymptomatic course underscores the limitations of symptom-based assessment alone and highlights the critical role of regular imaging surveillance in the detection of disease progression, development of aortic valve involvement, or secondary aortic pathology. Early identification of these changes is essential for timely intervention before irreversible myocardial or valvular damage occurs. Patients who remain asymptomatic but demonstrate progressive LVOT obstruction or deterioration of aortic valve function require close surveillance, as delayed intervention may increase the likelihood of long-term valvular dysfunction [50,51].

## 6. Hemodynamic Consequences and Natural History

SAS produces a fixed obstruction to left ventricular outflow, leading to characteristic and progressive hemodynamic alterations. The chronic pressure overload imposed by the LVOT obstruction results in concentric left ventricular hypertrophy, often with predominant interventricular septal thickening [5,13,19]. This remodeling may further exacerbate outflow tract narrowing, creating a vicious cycle of progressive obstruction and increasing systolic pressure gradients [13]. This compensatory response initially preserves systolic function and cardiac output; however, progressive hypertrophy ultimately leads to impaired diastolic relaxation and reduced ventricular compliance, which play a central role in symptom development [3,7,13].

The natural history of SAS differs importantly between pediatric and adult populations. In children, progression of LVOT obstruction may be relatively rapid and difficult to predict, particularly during periods of somatic growth and in the presence of unfavorable LVOT geometry or associated congenital lesions [5,13,14,15]. In contrast, adult cohorts with discrete SAS generally demonstrate substantially slower hemodynamic progression. Oliver et al. reported a relatively slow rate of progression of both obstruction and aortic regurgitation in adults [3], while the multicenter study by van der Linde et al. similarly demonstrated that progression of discrete SAS in adulthood is usually slow, although patients with associated congenital heart disease may experience more rapid progression [4]. Thus, the progressive nature of SAS should not be interpreted as implying a uniform rate of deterioration across age groups, and surveillance and intervention strategies should be individualized accordingly.

In isolated SAS, systolic function is usually preserved until advanced stages, whereas diastolic dysfunction develops earlier and contributes to exertional dyspnea and exercise intolerance [3,13,52,53]. The rate of progression is variable and unpredictable in children but generally slower in adults, although progression remains continuous in both populations [3,13,14,15]. Higher preoperative LVOT gradients have consistently been associated with more aggressive disease progression and an increased likelihood of future reintervention, emphasizing the importance of careful longitudinal assessment even in clinically stable patients [54,55,56,57].

Aortic valve involvement represents one of the most clinically relevant consequences of SAS. Chronic exposure of the aortic cusps to high-velocity, turbulent systolic jets results in progressive leaflet thickening, reduced mobility, and eventual aortic regurgitation (AR) [3,5,13,21]. In addition, fibromuscular tissue from the subaortic membrane may extend toward the aortic annulus, further impairing valve function [23]. Aortic regurgitation develops in up to 65% of patients and frequently persists or progresses even after surgical relief of the subvalvular obstruction [3,13,23]. Increasing LVOT gradients are associated with greater severity of regurgitation [3,15]. In advanced cases, concomitant aortic valve repair or replacement may be required at the time of surgical correction [3,5,13,37]. Long-term follow-up studies have demonstrated that progression of aortic regurgitation may continue despite successful relief of the LVOT obstruction, suggesting that irreversible leaflet injury may already be present at the time of surgery [50,54].

Post-stenotic dilatation of the ascending aorta is another important hemodynamic consequence and is attributed to abnormal flow patterns and increased wall shear stress distal to the obstruction [7,19,52]. This finding may progress independently of LVOT gradient severity and has important implications for surgical planning, as concomitant aortic replacement may be required.

The combined effects of pressure overload from LVOT obstruction and volume overload from AR significantly increase myocardial oxygen demand, while reduced aortic diastolic pressure impairs coronary perfusion [13,37,52]. This imbalance predisposes the hypertrophied myocardium to subendocardial ischemia and may contribute to angina, arrhythmias, and, in advanced cases, ventricular dysfunction. Persistent ventricular remodeling may also contribute to subtle impairment of myocardial mechanics despite preserved left ventricular ejection fraction, supporting the value of long-term functional assessment after surgical correction [58,59].

Overall, the natural history of SAS is characterized by gradual but relentless progression rather than spontaneous stabilization [9,13,30]. Without timely intervention, persistent LVOT obstruction and its secondary consequences may lead to irreversible myocardial remodeling, progressive valvular dysfunction, and adverse long-term outcomes, underscoring the importance of regular follow-up and appropriately timed surgical treatment [3,48]. Patients with a bicuspid aortic valve, younger age at operation, complex left-sided cardiac lesions, or high preoperative pressure gradients appear to be at particularly high risk for recurrence and warrant closer postoperative surveillance [56,57,58,59,60,61].

## 7. Diagnostic Evaluation

**Figure 4:**
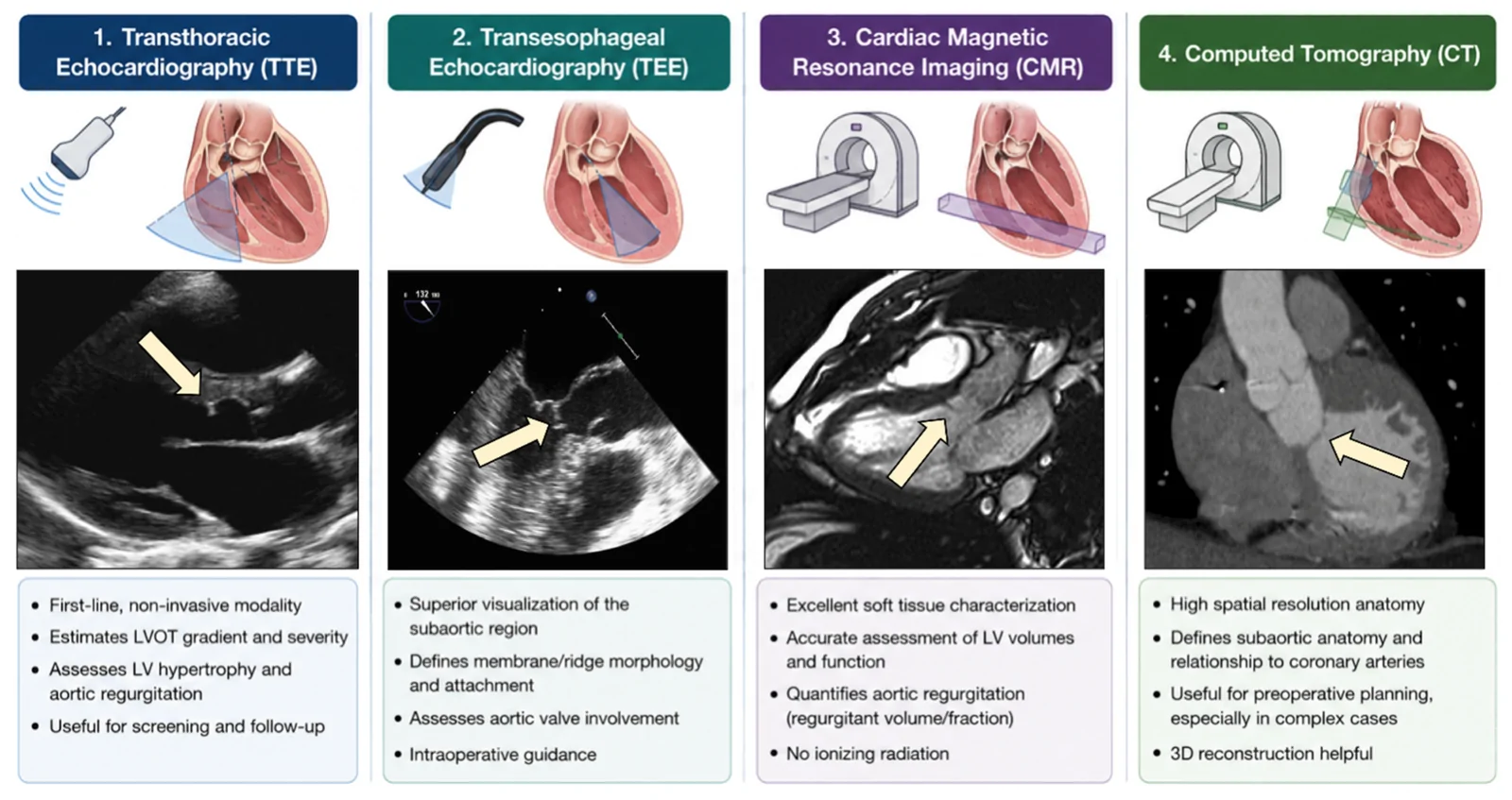
Multimodality imaging in subvalvular aortic stenosis. Representative examples of transthoracic echocardiography, transesophageal echocardiography, cardiac magnetic resonance imaging, and computed tomography illustrating their complementary roles in the diagnosis and anatomical characterization of subvalvular aortic stenosis. While echocardiography remains the first-line imaging modality for assessing lesion morphology and hemodynamic severity, cardiac magnetic resonance imaging and computed tomography provide additional anatomical detail and facilitate preoperative planning, particularly in complex cases.

Echocardiography represents the diagnostic cornerstone for the evaluation of SAS and provides comprehensive information regarding both anatomical and functional disease severity. Transthoracic echocardiography enables detailed assessment of LVOT gradients, left ventricular hypertrophy, lesion morphology, aortic and mitral valve integrity, post-stenotic dilatation of the ascending aorta, and the presence of associated congenital cardiac anomalies [13,52]. Doppler interrogation is essential for quantifying the degree of obstruction and monitoring disease progression over time. The principal imaging modalities currently used for the diagnosis and preoperative assessment of subvalvular aortic stenosis are summarized in Figure 4, highlighting their complementary roles in anatomical characterization, hemodynamic assessment, and surgical planning. The principal imaging modalities currently used in the evaluation of subvalvular aortic stenosis, together with their respective strengths, limitations, and clinical applications, are summarized in Table 2.

Advanced imaging techniques, including two-dimensional and three-dimensional echocardiography, further enhance spatial resolution and allow more precise characterization of the subaortic lesion, its extent, and its relationship to adjacent structures, thereby facilitating surgical planning [52,62,63]. Three-dimensional imaging is particularly useful in complex or atypical anatomical variants and in cases with concomitant valvular involvement. Three-dimensional echocardiography also improves visualization of the spatial relationship between the subaortic membrane, the aortic valve, and the mitral valve apparatus, facilitating accurate assessment of lesion complexity before surgery [17].

Specific echocardiographic windows play an important role in lesion visualization. Parasternal long-axis and subcostal views are especially helpful in determining the distance between the subaortic membrane and the aortic valve, a factor that has been associated with disease progression and risk of recurrence [5,13,37]. A membrane-to-aortic valve distance of less than 5 mm has been associated with a significantly increased likelihood of recurrent LVOT obstruction following surgical resection [56]. In cases where pronounced septal hypertrophy obscures the presence of a discrete membrane, transesophageal echocardiography offers superior diagnostic accuracy and is invaluable for differentiating SAS from hypertrophic septal obstruction or other causes of LVOT narrowing [3]. Intraoperative transesophageal echocardiography is also essential for confirming complete relief of the obstruction and assessing residual valvular dysfunction.

Cardiac magnetic resonance imaging provides complementary anatomical and functional information and is particularly useful for comprehensive preoperative evaluation. It allows accurate assessment of ventricular volumes and function, precise delineation of LVOT anatomy, and evaluation of associated aortic pathology without exposure to ionizing radiation [7,52]. Cardiac computed tomography offers excellent spatial resolution for evaluating LVOT morphology, aortic root anatomy, coronary anatomy, and ascending aortic pathology, making it particularly valuable for operative planning in adults with complex anatomy or suspected concomitant aortic disease [17,64,65].

Electrocardiography commonly reveals signs of left ventricular hypertrophy in approximately 50–80% of patients, reflecting chronic pressure overload [13]. Although cardiac catheterization is not routinely required for the diagnosis of isolated SAS, it may be indicated in adult patients as part of preoperative assessment to exclude significant coronary artery disease. Furthermore, despite the rarity of anomalous coronary artery origins in SAS, coronary angiography can aid in identifying congenital coronary anomalies that may influence surgical strategy and perioperative risk. A multimodality imaging approach combining echocardiography, CT, and cardiac magnetic resonance imaging provides the most comprehensive anatomical and functional evaluation in patients with complex SAS and associated congenital or aortic abnormalities [17].

## 8. Therapeutic Strategies and Surgical Indications

Medical therapy does not modify the natural history of SAS and therefore has no role in the long-term management of asymptomatic patients or in preventing disease progression. Although SAS is a progressive disorder, its rate of progression and eventual need for intervention vary considerably according to age, anatomical phenotype, hemodynamic severity, and associated cardiac abnormalities. Progression may be more pronounced and less predictable in pediatric patients, whereas adults with discrete SAS often demonstrate a slower clinical course and may remain stable under appropriate surveillance for prolonged periods [3,4,13]. Consequently, surgical intervention should not be considered inevitable in all patients but should be guided by symptoms, severity and progression of LVOT obstruction, ventricular response, aortic valve involvement, and the presence of associated cardiac lesions. Surgical resection remains the gold standard treatment and encompasses a spectrum of procedures ranging from simple excision of a discrete subaortic membrane to more extensive fibromuscular resection combined with septal myectomy or myomectomy, depending on the morphology and extent of the lesion [3,5,13,37]. The extent of surgical resection should be tailored to the underlying anatomical substrate, as isolated membrane excision is appropriate for discrete lesions, whereas diffuse or tunnel-type obstruction often requires more extensive enlargement of the LVOT [27].

The optimal timing of surgical intervention remains a subject of ongoing debate. Early surgery may theoretically limit progressive left ventricular hypertrophy, reduce the risk of aortic valve damage, and prevent adverse ventricular remodeling. However, these potential benefits must be carefully weighed against the well-documented risk of postoperative recurrence, the need for late reoperation, and the possibility of worsening or newly developed aortic regurgitation following relief of the obstruction [66,67,68,69,70]. Consequently, surgical decision-making must be individualized, taking into account patient age, symptom burden, LVOT gradient, rate of progression, and associated cardiac abnormalities. An individualized operative strategy based on LVOT anatomy, severity of obstruction, and associated valve abnormalities has been associated with favorable long-term outcomes across different anatomical subtypes [52].

In children and adolescents, surgical intervention is generally recommended in symptomatic patients with hemodynamically significant LVOT obstruction, particularly when the peak instantaneous Doppler gradient is ≥50 mmHg or the mean Doppler gradient is ≥30 mmHg. Symptoms such as exertional dyspnea, angina, or syncope further support intervention, while lower gradients may warrant consideration of surgery when accompanied by progressive aortic regurgitation, left ventricular hypertrophy or dysfunction, or documented progression of obstruction [7,13].

Surgical correction of SAS requires cardiopulmonary bypass and cardioplegic arrest. The subaortic membrane is most commonly approached via a transaortic route, allowing direct visualization of the LVOT after gentle retraction of the aortic valve cusps. Complete and meticulous resection of the membrane is essential to minimize the risk of recurrence. When significant septal hypertrophy contributes to obstruction, adjunctive septal myectomy may be performed to further widen the LVOT. Routine septal myectomy, however, has not consistently been shown to reduce recurrence rates compared with membrane excision alone and should therefore be performed selectively according to anatomical findings rather than as a universal adjunct [71,72]. Concomitant aortic valve repair should be pursued in cases of cusp prolapse or mild regurgitation, whereas valves with advanced calcification, fibrosis, or dysplasia generally require replacement. When moderate or severe aortic regurgitation is present, concomitant aortic valvuloplasty may improve postoperative valve function while avoiding valve replacement in selected patients [27].

In critically ill infants or patients who are poor candidates for immediate open-heart surgery, percutaneous balloon dilation may be used as a palliative measure to temporarily reduce LVOT gradients and stabilize hemodynamics [13]. Although this approach does not provide definitive treatment and is associated with a high rate of restenosis, it may allow time for somatic growth or clinical optimization prior to surgical repair. More complex anatomical variants, such as tunnel-type SAS associated with a small LV–aortic junction, often require more aggressive surgical strategies, including the Konno or modified Konno procedure, to adequately enlarge the LVOT and achieve durable relief of obstruction [7,13,37]. Patients with concomitant ascending aortic aneurysm or significant structural aortic valve disease should undergo simultaneous correction of all clinically relevant lesions during the index operation whenever feasible, thereby avoiding staged procedures and optimizing long-term outcomes [61]. Overall operative mortality remains low, and contemporary surgical series continue to demonstrate excellent early postoperative outcomes despite the anatomical complexity of many cases [33].

**Figure 5:**
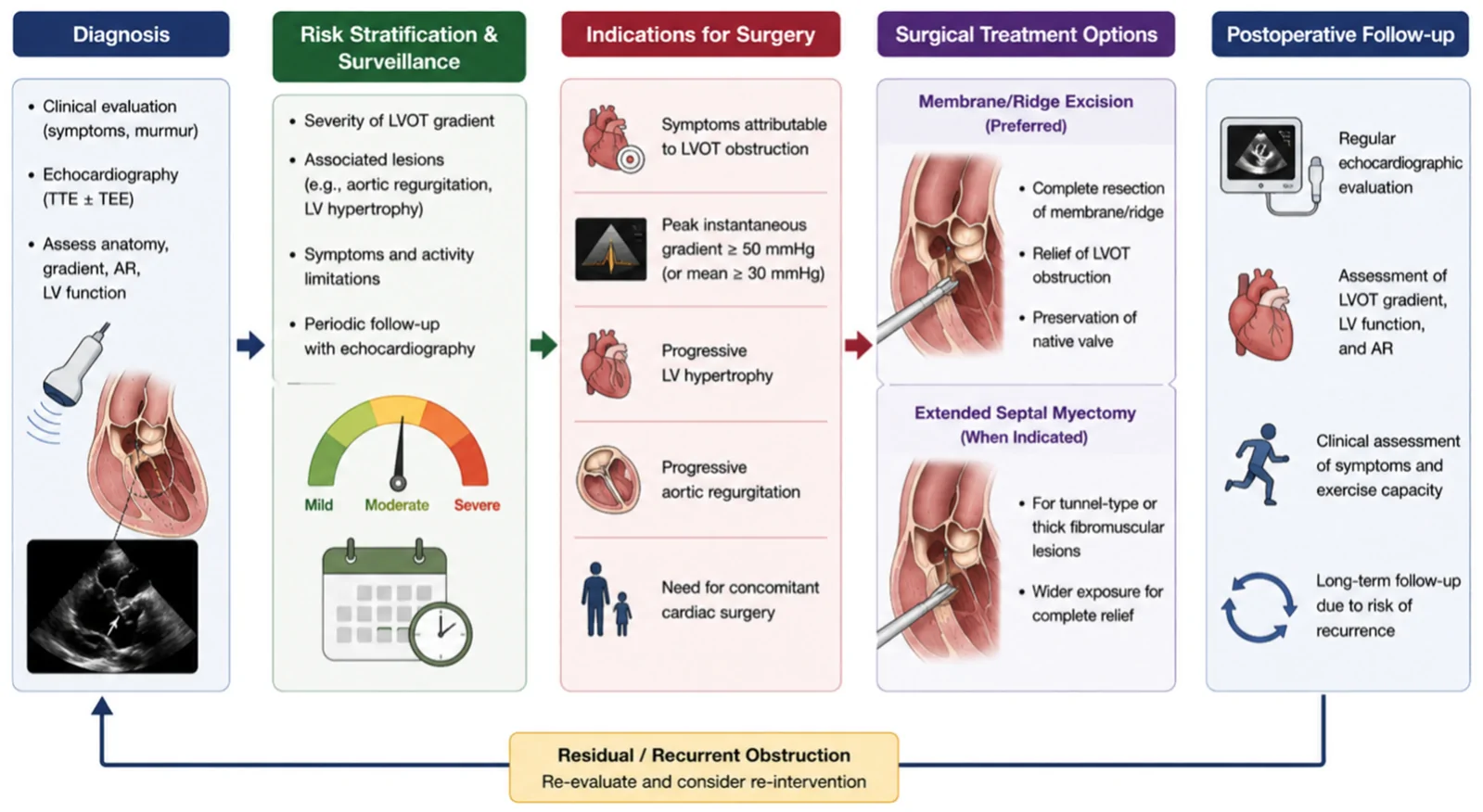
Management strategy for subvalvular aortic stenosis. Proposed clinical pathway for the evaluation and management of patients with subvalvular aortic stenosis, including diagnosis, risk stratification, indications for surgical intervention, operative treatment, and long-term postoperative surveillance. Regular follow-up is essential because of the progressive nature of the disease and the potential for residual or recurrent left ventricular outflow tract obstruction.

Current recommendations regarding surgical intervention are derived from professional society guidance, observational studies, and longitudinal surgical series; however, the strength and specificity of the supporting evidence vary considerably across clinical scenarios. Current indications for surgical intervention are summarized in Table 3, while the principal factors associated with postoperative recurrence of subvalvular aortic stenosis are presented separately in Table 4. The overall diagnostic and therapeutic approach to patients with subvalvular aortic stenosis is summarized in Figure 5, illustrating the pathway from initial diagnosis and risk stratification to surgical decision-making and long-term postoperative follow-up.

Supporting evidence refers to the principal sources informing each recommendation, including contemporary international guidelines, observational studies, and expert consensus documents.

- High–Recommendations consistently supported by major international guidelines and multiple observational studies.
- Moderate–Recommendations supported primarily by observational studies and expert consensus.
- Low–Recommendations based mainly on limited observational evidence or expert opinion.

Note: The qualitative categories “High”, “Moderate”, and “Low” represent the authors’ narrative assessment of the consistency, quantity, and clinical applicability of the available evidence and should not be interpreted as formal guideline-defined levels of evidence. Recommendations from professional society guidelines were considered where applicable and are cited individually in the accompanying text.

## 9. Outcomes, Recurrence, and Long-Term Follow-Up

Long-term survival following surgical treatment of SAS is generally excellent; however, recurrence of LVOT obstruction remains a well-recognized limitation of surgical therapy and mandates lifelong follow-up [52]. Recurrence may occur due to incomplete initial resection, continued abnormal flow dynamics, or persistent underlying LVOT geometry that promotes renewed fibromuscular proliferation. For this reason, close postoperative surveillance is essential, particularly in pediatric patients, who should be monitored at regular intervals—often every six months—to detect early increases in LVOT gradients and associated valvular changes. Contemporary surgical series generally demonstrate favorable freedom from reoperation after primary SAS resection, although reported estimates vary according to patient age, anatomical substrate, operative strategy, endpoint definition, and duration of follow-up [54]. Importantly, crude reoperation rates and actuarial freedom-from-reoperation estimates should not be interpreted interchangeably, particularly when follow-up duration differs substantially between cohorts.

In a contemporary series of 120 patients undergoing primary surgical correction of SAS, Bandara et al. reported freedom from LVOT reintervention of 99%, 94%, 93%, and 90% at 1, 3, 5, and 10 years, respectively, over a median follow-up of 13 years [54]. In a cohort of 84 patients undergoing isolated subaortic membrane resection, Binsalamah et al. reported that 12 patients (14%) required one reoperation and one patient required two reoperations during a median follow-up of 9.3 years, with a median time to first reoperation of 4.6 years [59]. In a separate pediatric cohort of 104 patients undergoing primary resection of discrete SAS, Mukadam et al. reported repeat resection in 9 patients (8.4%) during a median follow-up of 8.5 years, with actuarial freedom from repeat resection of 100%, 94%, and 82% at 1, 5, and 10 years, respectively [73]. Selected contemporary surgical series illustrating the variability in recurrence and reintervention according to cohort characteristics, outcome definition, and duration of follow-up are summarized in Table 5. These estimates should not be regarded as contradictory, as crude reoperation rates and actuarial freedom-from-reoperation estimates represent different outcome measures and are influenced by cohort characteristics and duration of follow-up.

Pacemaker implantation was required in 2.9% of cases, reflecting the proximity of the resection area to the cardiac conduction system [73,74]. Higher recurrence rates have been reported in patients with underlying genetic or syndromic conditions, suggesting that intrinsic anatomical or tissue-related factors may contribute to disease persistence [73]. Independent predictors of recurrence include younger age at operation, higher preoperative LVOT gradients, shorter membrane-to-aortic valve distance, bicuspid aortic valve, and associated left-sided obstructive lesions [54,56,60]. Collectively, these findings demonstrate that recurrent obstruction may continue to emerge over extended follow-up despite favorable early surgical results, reinforcing the need for lifelong postoperative surveillance.

Familial forms of SAS, including those described in association with Shone syndrome, have been reported, although robust data regarding optimal treatment strategies and long-term outcomes in these patients remain limited [75,76,77]. These cases further support the need for individualized management and extended follow-up.

Postoperative complications reported in the literature include conduction abnormalities such as bundle branch block or complete heart block, iatrogenic ventricular septal defect, mitral valve injury, progression or persistence of aortic regurgitation, incomplete relief or recurrence of LVOT obstruction, and infective endocarditis [13,52]. Although surgical relief effectively reduces LVOT obstruction, progression of aortic regurgitation may still occur during long-term follow-up, particularly in patients with pre-existing leaflet abnormalities or advanced valvular involvement at the time of surgery [50]. Functional recovery and long-term quality of life are closely linked to the degree of residual obstruction and valvular dysfunction. Most patients experience sustained symptomatic improvement and preservation of ventricular function after successful surgery, although subtle impairment of myocardial performance may persist despite normal left ventricular ejection fraction [58]. Consequently, recommendations regarding physical activity should be individualized based on postoperative hemodynamic findings, with avoidance of strenuous exercise and competitive sports generally advised in patients with residual gradients or valvular disease [13].

Recurrent obstruction should not automatically be attributed to incomplete primary resection, as ongoing remodeling of the LVOT and patient-specific anatomical characteristics may contribute to late fibromuscular proliferation despite technically adequate surgery [54,55,56,57]. Long-term outcomes are optimized by complete excision of the obstructive lesion, individualized management of associated aortic valve and ascending aortic pathology, and structured lifelong follow-up with periodic imaging [61,62].

## 10. Limitations

This review has several limitations that should be acknowledged. First, it represents a narrative synthesis of the available literature rather than a systematic review or meta-analysis; therefore, the selection of included studies may be subject to inherent selection bias. Second, much of the current evidence regarding subvalvular aortic stenosis is derived from retrospective observational studies and relatively small patient cohorts, which may limit the strength of available evidence and the generalizability of reported outcomes. In addition, heterogeneity among studies with respect to patient populations, imaging techniques, surgical approaches, and follow-up duration makes direct comparison of results challenging. These limitations highlight the need for larger prospective studies and standardized reporting to better define optimal management strategies and long-term outcomes in patients with subvalvular aortic stenosis.

## 11. Conclusions

Subvalvular aortic stenosis is a complex and progressive cause of left ventricular outflow tract obstruction with highly variable clinical presentation and natural history. Although often classified as a congenital lesion, accumulating evidence supports its dynamic and, in many cases, acquired behavior driven by abnormal LVOT anatomy and disturbed flow patterns. Careful and comprehensive imaging—centered on echocardiography and complemented by advanced modalities—is essential for accurate diagnosis, assessment of associated lesions, and optimal surgical planning. Surgical resection remains the definitive treatment and should be performed when clear hemodynamic or clinical indications are present, with particular attention to complete membrane excision, management of associated aortic valve pathology, and ascending aortic involvement when indicated. Despite excellent long-term survival, the risk of recurrence and progressive aortic regurgitation necessitates lifelong follow-up. Future research focusing on improved risk stratification, earlier identification of patients at risk for progression, and refinement of surgical strategies may further optimize outcomes in this challenging condition.

## 12. Key Points

- Subvalvular aortic stenosis is a progressive form of left ventricular outflow tract obstruction rather than a purely congenital fixed lesion.
- Transthoracic echocardiography remains the cornerstone of diagnosis and long-term surveillance.
- Progressive aortic regurgitation is an important consequence of untreated disease.
- Surgical resection remains the treatment of choice for hemodynamically significant obstruction.
- Despite excellent early outcomes, recurrence remains an important challenge requiring lifelong follow-up.
- Advanced multimodality imaging facilitates surgical planning in anatomically complex cases.

## Figures and Tables

**Table 1:** Morphological classification and clinical characteristics of subvalvular aortic stenosis.

Morphological Type	Anatomical Characteristics	Typical Age at Presentation	Hemodynamic Severity	Associated Findings	Preferred Surgical Approach	Risk of Recurrence *
Discrete subaortic membrane	Thin fibrous membrane immediately beneath the aortic valve, usually attached to the interventricular septum	Childhood to adolescence	Mild to moderate	Mild aortic regurgitation may develop with disease progression	Complete membrane excision	Moderate
Fibromuscular ridge	Thickened fibromuscular shelf with variable muscular component	Childhood or young adulthood	Moderate	Progressive LV hypertrophy, possible aortic regurgitation	Membrane excision with limited septal myectomy when indicated	Moderate
Tunnel-type subaortic stenosis	Long, diffuse fibromuscular narrowing of the LVOT	Childhood	Moderate to severe	Marked LV hypertrophy, higher transvalvular gradients	Extended septal myectomy with complete relief of obstruction	High
Complex subaortic stenosis	Diffuse LVOT obstruction associated with additional congenital cardiac abnormalities	Variable	Variable, frequently severe	Bicuspid aortic valve, ventricular septal defect, coarctation of the aorta, Shone complex, or other congenital lesions	Individualized surgical correction addressing all associated lesions	High

LVOT: left ventricular outflow tract. * Recurrence risk refers to the relative likelihood of recurrent LVOT obstruction reported in the literature and is expressed qualitatively (low, moderate, or high) because published recurrence rates vary considerably according to patient age, anatomical subtype, surgical technique, duration of follow-up, and study design.

**Table 2:** Multimodality imaging in the evaluation of subvalvular aortic stenosis.

Imaging Modality	Principal Role	Main Strengths	Limitations	Typical Clinical Indications
Transthoracic echocardiography (TTE)	First-line diagnostic examination	Widely available; evaluates LVOT morphology, Doppler gradients, left ventricular hypertrophy, aortic regurgitation, and associated congenital lesions	Image quality may be limited by poor acoustic windows or obesity	Initial diagnosis, disease severity assessment, and routine follow-up
Transesophageal echocardiography (TEE)	Detailed anatomical evaluation	Superior visualization of the subaortic membrane, LVOT anatomy, aortic valve, and mitral valve apparatus; valuable intraoperatively	Semi-invasive procedure requiring sedation	Inconclusive TTE, complex anatomy, preoperative assessment, and intraoperative guidance
Cardiac magnetic resonance imaging (CMR)	Comprehensive anatomical and functional assessment	Excellent evaluation of ventricular volumes and function, LVOT anatomy, myocardial tissue characterization, and associated cardiovascular abnormalities without ionizing radiation	Limited availability, longer acquisition time, contraindications in selected patients	Complex anatomy, inconclusive echocardiography, assessment of ventricular remodeling, and preoperative planning
Computed tomography (CT)	High-resolution anatomical imaging	Excellent spatial resolution for assessment of LVOT anatomy, aortic root, ascending aorta, and coronary arteries; useful three-dimensional reconstruction	Exposure to ionizing radiation and iodinated contrast agents	Surgical planning, evaluation of associated aortic pathology, and coronary artery assessment in adult patients

LVOT: left ventricular outflow tract; TTE: transthoracic echocardiography; TEE: transesophageal echocardiography; CMR: cardiac magnetic resonance imaging; CT: computed tomography.

**Table 3:** Clinical indications for surgical intervention in subvalvular aortic stenosis.

Clinical Scenario	Recommended Management	Clinical Rationale	Authors’ Qualitative Assessment of Supporting Evidence *	Supporting Evidence
Symptomatic patient with significant LVOT obstruction	Surgical intervention	Relief of obstruction and improvement of symptoms	High	ACC/AHA and ESC Guidelines; consistent observational studies
Peak instantaneous Doppler gradient ≥ 50 mmHg (or mean gradient ≥ 30 mmHg)	Surgical intervention generally recommended	Prevent progression of LVOT obstruction and adverse cardiac remodeling	High	ACC/AHA and ESC Guidelines
Progressive or significant aortic regurgitation	Surgical intervention	Prevent irreversible aortic valve damage and preserve valve function	Moderate	ESC Guidelines; observational studies
Progressive left ventricular hypertrophy or declining ventricular function	Consider surgical intervention	Reduce chronic pressure overload and adverse ventricular remodeling	Moderate	Observational studies and expert consensus
Rapid increase in LVOT gradient during serial follow-up	Consider earlier intervention	Suggests progressive disease and increasing hemodynamic burden	Moderate	Longitudinal observational studies
Planned surgery for associated congenital cardiac disease	Concomitant membrane resection when appropriate	Avoid future reoperation and persistent LVOT obstruction	High	ACC/AHA and ESC Guidelines; expert consensus

* Level of evidence reflects the overall strength and consistency of the available published literature supporting each recommendation and is expressed qualitatively (High, Moderate, or Low) because robust randomized clinical trials are lacking in subvalvular aortic stenosis. Note: The qualitative categories “High,” “Moderate,” and “Low” represent the authors’ narrative assessment of the consistency, quantity, and clinical applicability of the available evidence and should not be interpreted as formal guideline-defined levels of evidence. Recommendations from professional society guidelines were considered where applicable and are cited individually in the accompanying text.

**Table 4:** Factors associated with postoperative recurrence of subvalvular aortic stenosis.

Risk Factor	Proposed Mechanism	Clinical Implication
Young age at operation	Ongoing somatic growth and continued LVOT remodeling	Increased need for long-term surveillance
High preoperative LVOT gradient	More advanced disease	Higher recurrence risk
Tunnel-type subaortic stenosis	Diffuse and extensive obstruction	Greater technical complexity and recurrence
Incomplete resection of the membrane	Residual obstructive tissue	Persistent or recurrent obstruction
Residual postoperative LVOT gradient	Incomplete relief of obstruction	Predictor of earlier recurrence
Small aortic annulus or complex LVOT anatomy	Persistent abnormal flow dynamics	Increased likelihood of recurrent obstruction
Associated congenital cardiac abnormalities	More complex anatomical substrate	Greater probability of repeat intervention

LVOT: left ventricular outflow tract. Note: Recurrence risk is expressed qualitatively because reported recurrence rates vary according to patient age, anatomical phenotype, operative technique, outcome definition, and duration of follow-up.

**Table 5:** Selected contemporary surgical series reporting recurrence and reintervention following surgical treatment of subvalvular aortic stenosis. The table summarizes representative studies with intermediate- to long-term follow-up, highlighting differences in study population, sample size, follow-up duration, outcome definitions, and reported recurrence or reintervention rates. Differences between crude reoperation proportions and actuarial freedom-from-reintervention estimates should be considered when comparing outcomes across studies.

Study	Population/Surgical Cohort	Sample Size	Follow-Up	Endpoint	Principal Outcome
Bandara et al. [54]	Patients undergoing primary surgical correction of SAS; patients with major associated lesions such as aortic arch obstruction were excluded	120	Median 13 years (IQR 7–18)	Recurrence and LVOT reintervention	Recurrence occurred in 20 patients (18%); 15 patients underwent reintervention. Freedom from reintervention was 99%, 94%, 93%, and 90% at 1, 3, 5, and 10 years, respectively
Binsalamah et al. [59]	Patients undergoing resection of an isolated subaortic membrane; patients undergoing concomitant procedures were excluded	84	Median 9.3 years (IQR 0.6–22.5)	Reoperation following isolated subaortic membrane resection	12 patients (14%) required one reoperation, and 1 patient required two reoperations; median time to first reoperation was 4.6 years. Septal myectomy was associated with longer freedom from reoperation
Mukadam et al. [73]	Pediatric patients undergoing primary surgical resection of discrete SAS	104	Median 8.5 years (IQR 5.9–13.5)	Repeat resection for recurrent subaortic membrane	9 patients (8.4%) required repeat resection. Actuarial freedom from repeat resection was 100%, 94%, and 82% at 1, 5, and 10 years, respectively

Abbreviations: IQR, interquartile range; LVOT, left ventricular outflow tract; SAS, subvalvular aortic stenosis.

## Data Availability

No new data were created or analyzed in this study.
